# Consumption Curable; and the Manner in Which Nature as Well as Remedial Art Operates in Effecting a Healing Process in Cases of Consumption, &c.

**Published:** 1834-07-01

**Authors:** 


*Consumption Curable; and the Manner in which Nature as well as Remedial Art operates in effecting a Healing Process in Cases of Consumption, &c.* By Francis Hopkins Ramadge, m.d., f.l.s., Fellow of the Royal College of Physicians.— London, 1834. 8vo. pp. 168; four Plates.

Some well-meaning but mistaken persons have accused us of a bias in favour of the Fellows of the College of Physicians, and have thought that, in the debates which now convulse the medical republic, we have advocated with too much warmth the cause of those grasping monopolists, several of whom have actually seized upon places worth twenty pounds sterling per annum! Now, we plead not guilty of a bias, but guilty of a predilection. We have always been pleased with the favourite phrase of our old writers, " a grave and *learned* doctor of physic," and we have thought that those who have been hurried away from their books at fifteen or sixteen, and placed behind desks or counters to pick up knowledge by stealth or accident, were not quite so likely to maintain the ancient character of the profession, as those who have had unlimited opportunities of study, and have drunk deep from those fountains of learning which, for ages, all that is most honourable and most revered in England has combined to foster. This theory we were prepared, and are still prepared, to uphold by an appeal to facts; yet we fear that our adversaries (if critics so good-humoured as ourselves can have any,) will poke "Consumption Curable" in our faces, and will say " Here is a Fellow for you!" just as when the philosopher of old had unluckily defined man to be a biped without feathers, somebody thrust a cock stripped of his feathers into his school, and cried out " Here is Plato's man for you!" We trust, therefore, that we shall receive due credit for our candour in confessing that the work before us is of inconceivable poorness and flatness, and stands unrivalled for pompous imbecility and boastful nonsense. If we were thick-and-thin partisans of the Fellows, (which we are not,) our sole consolation would consist in the reflection, that, since the foundation of the College, there has been but one Ramadge; and that copies of this book, the few copies that are preserved, (for books of this kind have a wonderful tendency to grow scarce,) might be put up in glass bottles, as far greater curiosities than a scirrhous heart, or an acephalous fœtus.

We are afraid that, when we tell our readers how Dr. Ramadge proposes to cure phthisis, they will imagine that we are indulging in an agreeable embellishment, but we assure them that we never were more serious in our lives. The first *methodus medendi*, then, consists in producing an emphysematous state of the lungs by inhalation; and the second way is, to make the patient catch cold, as a catarrh is not only an immediate benefit, but is capable of effecting a lasting cure. And now for a few extracts, to justify us in our readers' eyes.

"There are but two modes by which we can hope to cure this disease; the one is by rendering it chronic, and the other by artificially enlarging those portions of the lungs which are pervious to air. In the first, we endeavour to effect an absence of constitutional disorder; but, after this change is effected, there may still remain for an indefinite period one or more cavities uncicatrised, with lining membrane, partly semi-cartilaginous, or of such condensed and insensible structure as to be productive of little inconvenience, if we except occasional cough, and some hæmorrhage occurring at long intervals, and rarely to a great extent. In the second there is produced what is invariably seen when nature or art has effected a cure, an enlargement of the vesicular structure of the lungs, and subsequently a gradual healing of the tuberculous excavations." (P. 88.)

"*Inhalation.* I am well aware that many objections may be started to this practice from prejudice, or inefficient observation. Strange to say, the principle, on which this mode of treatment operates beneficially, appears to me quite unknown to medical men. It is supposed that the inspiration of medicated vapours has in many instances proved useful by allaying cough, and by producing some healthy and unexplained change in diseased parts of the lungs, as well as on such adventitious surfaces as are formed after the softening, or discharge, of tuberculous matter. The permanent advantages which inhaling is capable of affording, I am convinced, have been very rarely witnessed by the generality of practitioners.

First, because the period, during which persons are directed to inhale, is generally too short to produce either a catarrhal, or an enlarged state of the lungs, one of which conditions is absolutely necessary in order to suspend, or cure consumption: and, secondly, the apparatuses employed for this purpose are not constructed scientifically, so as to facilitate those physical changes, which it is desirable the chest should undergo." (P. 97.)

"Neither perfect recovery, nor indeed exemption from the danger of relapse into a consumptive state, is found to occur, except in very rare instances, unless the pulmonary organs become naturally or artificially voluminous; which not unfrequently happens by the supervention of some catarrhal state of the larynx, trachea, or bronchial tubes. It is a most fortunate circumstance for some affection of this kind to occur early, as it never fails permanently to arrest this most fatal disorder. When the lower lobes of the lungs are entirely free from tuberculous matter, (which is often indisputably the case for a considerable period, unless there be strong hereditary predisposition;) and though there exist at the same time cavities in the superior part of one or both lungs, clearly indicated by perfect pectoriloquism, there is almost a never failing hope of recovery to be entertained, provided an emphysematous sound can be heard.

"In fact, I never knew a consumptive person who did not lose all his formidable symptoms and regain health, when an emphysematous, or a semi-asthmatic change had early taken place. And likewise, I never knew an individual to become consumptive who was a subject of chronic catarrh, or any species of asthma. It is from long consideration of these facts, that I interfere but little with any catarrhal inflammation, which may show itself in the midst of consumptive symptoms, for I well know that it will gradually supersede all these." (P. 100.)

"*Climate.* It has been seen from the preceding pages how much I am at variance with the common opinions entertained of phthisis, and to none am I more diametrically opposed than to those which respect climate. So far from sending a consumptive patient to the south of France or Italy, I should, if change be requisite, deem the climate of St. Petersburgh a thousand times more beneficial. In the latter case he has a chance of contracting catarrh, and of thus staying consumption; in the former, any catarrhal state which might exist would assuredly be fatally removed. My experience on this point is full and explicit; and I could substantiate it, were it requisite, at the close of a treatise, the scope of which has been to prove the true nature of this little understood malady, by numerous cases. So decided am I on this head, that I never admit into the Infirmary a phthisical patient with recent catarrh, because its wards are heated in winter-time so as to resemble a moderate summer temperature. In uniformity with these opinions, I feel no anxiety respecting consumptive patients being

5

kept scrupulously withindoors. Whenever the weather permits, they should be allowed to take an airing daily; but should not be suffered to remain so long as to be sensible of chilliness or cold." (P. 131.)

There is an appendix of thirteen cases; but, though we can afford room for one only, we will regale our readers with the titles of all, as they are pleasant enough in their way.

1. Supposed consumption cured by paracentesis.

2. Consumption cured by paracentesis. [The patient died, however.]

3. Consumption, in its very advanced stage, singularly arrested. [The patient died, in two months, of pleurisy.]

4. Pulmonary excavation discharging its contents by an opening made in the right side of the neck. [Patient died.]

5. Consumption cured by paracentesis.

6. Consumption cured by suddenly supervening empysema.

7. Consumption cured by neglect.

"I will venture one observation on this case, which, although it may appear harsh, regard for truth and the advancement of medical science compel me to make, namely, that had this person when reduced to a phthisical state recurred to medical advice, the probability is, the bronchial affection, which was his safeguard, would, as is but too frequently the practice, have been interfered with, its value being unknown to the profession, and his life consequently shortened by many years." (P. 156.)

8. Consumption spontaneously cured.

9. Remarkable influence of the protective effect of bronchial affection.

10. Complication of disease ending in phthisis.

11. The protecting influence of catarrh, supervening on consumption, exemplified.

12. Consumption cured by exposure to cold, and want of faith in medicine.

"Mr. D—, aged twenty-four, through irregular habits, had so materially impaired his constitution, as to fall in consequence into a decline. He of course availed himself of the benefit of medical advice, which produced no very visible amelioration of his state of health. Being naturally of active habits, he grew impatient of the confinement to which he was subjected, and, tempted by the return of spring, he suddenly deserted his heated apartment, and determined, since he concluded he must die, to die in the manner most agreeable to himself. Accordingly, he betook himself to his favorite sport of fishing. This was in the month of March, a period at which easterly winds are most prevalent. The worst consequence of this apparently rash exposure was, that, after a time, he caught cold, which, as it would appear, was confined to the trachea. His respiration was sensibly affected, and he laboured under a distressing fulness of the chest. He continued subject to this affection, with an apparent increase of the violence of his disorder; but he still rejected all care and medicine, and persevered in going out. After some period he began to exhibit signs of amendment: he gradually lost his emaciated appearance, acquired flesh and bodily vigor; but was much annoyed by wheezing of the chest, and loud rattle in his throat. He had remained in this state for some months when he applied to me. On examination of his chest, and hearing a detail of his complaints, not only from himself, but from the gentleman under whose care he had previously been, I at once perceived that he was indebted for his recovery from consumption to this catarrhal state of the trachea. I may here observe, that recoveries of this kind are more frequent among the lower than the other classes of the community, owing, doubtless, to what may at first appear a misfortune, but is, to the consumptive patient, in numerous instances, a blessing,—exposure to cold!" (P. 164.)

13. Case of consumption relieved by inhalation, and detailed by the patient himself.

"Ohe jam satis est!" our readers will exclaim. By no means; we have a titbit in store for you: it is not about physic, but about law; Dr. Ramadge, you know, is fortunate in both.

"It has been my misfortune to witness, in the course of my practice, but too many afflicting instances of the maladies engendered 'by a mind diseased.' Well has the poet of Nature remarked this, amongst his aggregate of human ills, when he mentions 'the law's delay,' than which I know not a greater destroyer of peace of mind, and with it of the body's health. We are accustomed to look back with horror on the proceedings recorded by historians as having taken place in that iniquitous court, which went under the name of the Star Chamber. How then, judging by analogy, will our posterity execrate the records left them of the practice of the Court of Chancery! They will there read a piteous tale of justice withheld, of hope, the brightest boon of heaven, extended and protracted, until, to look forward, is to exclaim, with *Lear*, 'Oh! that way madness lies!' The ghastly train of diseases, consumption, cancer, and other fell destroyers of the human race, which I have seen brought on the wretched victims of the procrastination, until recently the characteristic of this court, leads me, in common with the general voice, to reflect with gratitude on the change that has been effected by the wise energy of one master-spirit. Had the reforms introduced by him been adopted even a few years sooner, how many a fair fabric of human happiness would have been spared that now lies dismantled and overthrown!" (P. 86.)

It is needless to repeat our opinion of this work. The most doubting will cease for a time to hesitate; professed panegyrists will be unable to praise; and Dr. Ramadge's work will at least have the merit, if it has no other one, of making critics unanimous.

